# Leveraging pediatric PROMIS item banks to assess physical functioning in children at risk for severe functional loss

**DOI:** 10.1186/s41687-017-0011-8

**Published:** 2017-11-20

**Authors:** Angie Mae Rodday, Robert J. Graham, Ruth Ann Weidner, Nan E. Rothrock, Darren A. Dewalt, Susan K. Parsons

**Affiliations:** 1 Institute for Clinical Research and Health Policy Studies, Tufts Medical Center, Department of Medicine, Tufts University School of Medicine, 800 Washington St, Box 345, Boston, MA 02111 USA; 2 Department of Anesthesiology, Perioperative and Pain Medicine, Division of Critical Care Medicine, Boston Children’s Hospital, Department of Anesthesia, Harvard Medical School, 300 Longwood Avenue, Bader 629, Boston, MA 02115 USA; 3 Institute for Clinical Research and Health Policy Studies, Tufts Medical Center, 800 Washington St, Box 345, Boston, MA 02111 USA; 4 Department of Medical Social Sciences, Northwestern University Feinberg School of Medicine625, North Michigan Avenue, Suite 2700, Chicago, IL 60611 USA; 5 Division of General Medicine and Clinical Epidemiology, University of North Carolina at Chapel Hill, 5041 Old Clinic Building, CB 7110, Chapel Hill, NC 27599 USA; 6 Institute for Clinical Research and Health Policy Studies, Tufts Medical Center, Department of Medicine, Tufts University School of Medicine, 800 Washington St, Box 345, Boston, MA 02111 USA

**Keywords:** Pediatrics, Physical functioning, Health-related quality of life, PROMIS, Muscular disorders, Neuromuscular disorders

## Abstract

**Background:**

Pediatric neuromuscular illnesses often result in decreased health-related quality of life (HRQL), notably in physical functioning. Generic HRQL measures have been developed for use in general populations, but may not adequately assess patients with severe functional loss. To address this measurement gap, we created two custom parent-proxy physical functioning short forms for use among children at risk for low levels of functioning, using pediatric Patient Reported Outcomes Measurement Information System (PROMIS) item banks for Upper Extremity and Mobility.

**Methods:**

Two custom short forms from PROMIS Upper Extremity (13 items) and Mobility (13 items) parent-proxy item banks were created and administered to parents of children (ages 5 – 22 years) enrolled in an integrated care program for management of chronic respiratory insufficiency, largely due to neuromuscular illnesses. Standardized PROMIS T-scores have a mean of 50 (SD = 10); higher scores indicate better functioning. Physicians rated clinical severity. Single proxy-rated items on mental and physical health from the Child Health Rating Inventories (CHRIs) global health scale were completed by parents*.* Psychometric properties, including known groups comparisons, were explored.

**Results:**

Fifty-seven parents completed the parent-proxy custom PROMIS short forms. The mean Upper Extremity T-score was 21 (SD = 13); the mean Mobility T-score was 22 (SD = 11). Some participants scored at the measurement floor; two items on assistive devices did not perform well in this sample and were excluded from the Mobility T-score. Known groups comparisons showed that those with lower clinical severity had better median Upper Extremity (22 vs. 14, *p* < 0.001) and Mobility (28 vs. 16, *p* = 0.004) function than those with worse clinical severity. Both Upper Extremity and Mobility T-scores were higher in the subgroups defined by better physical and mental health, as measured by the CHRIs.

**Conclusions:**

Upper Extremity and Mobility T-scores were nearly three standard deviations below the PROMIS pediatric calibration population mean. Preliminary psychometrics demonstrated the potential to more accurately measure lower physical functioning using items from PROMIS item banks. However, some participants scored at the measurement floor despite targeting items at the lower end of the scale. Further short form refinement, enrichment of the item banks, and larger-scale field testing are needed.

## Background

Chronic illnesses of childhood, including the muscular dystrophies (MD), spinal muscular atrophy (SMA), skeletal dysplasias, and spinal cord injuries, may result in decreased health-related quality of life (HRQL), notably in the domain of physical health [1, 2]. The physical functioning component of physical health can be measured using clinical assessments and/or by self- (or parent-proxy) report questionnaires. Typically, self- or proxy ratings measure physical functioning using items or questions assessing varying levels of difficulty from mild to strenuous. Historically, HRQL measures included a fixed number of items within a given domain to capture a broad range of functioning for the majority of the population. However, many of these scales include items about walking, exercising, and lifting [3–5], which may not adequately capture physical function among patients with severe functional loss or allow for discrimination across patients or within patients over time [6]. Thus, assessing physical function in a population that is expected to have low functioning using existing measures has proven challenging.

In the clinical setting, several measures are used among those with limitations due to neuromuscular disorders, including the Performance of the Upper Limb (PUL) [7], the Brooke Upper Extremity Scale [8], the North Star Ambulatory Assessment for ambulatory children [9], the Hammersmith Functional Motor Scale (used in patients with SMA) [10], and the Egen Klassifikation (EK) scale for non-ambulatory children [11]. These measures are typically completed by a trained clinician (e.g., physiotherapists), as part of the clinic visit. Disease-specific HRQL instruments, such as the Pediatric Quality of Life Inventory (PedsQL™) Neuromuscular module rely on self- (or parent-proxy) report to measure areas of HRQL specifically affected by a given disease or condition, but only allow comparison within the disease group. In some instances, such comparisons within diseases or conditions are important or desired. However, our goal was to explore the use of a generic tool, rather than a clinical assessment or disease-specific instrument, to collect and compare HRQL domain scores across a range of different diagnoses and ages, and particularly in children with low physical functioning. Using a generic tool is important because each of these illnesses is relatively rare. Secondary complications, such as respiratory insufficiency, are often managed in multi-disciplinary clinics, spanning various underlying conditions. In addition, unlike clinical assessments, HRQL instruments do not require clinician involvement, so they can be administered remotely by self- or parent-proxy report, possibly between or during clinical visits, and can provide unique information about the patient experience not available from clinical assessments.

The Patient-Reported Outcomes Measurement Information System (PROMIS™), created in 2004 as part of a trans-National Institutes of Health (NIH) initiative to enhance the tools of clinical research, offers a new and improved method for generic HRQL assessment across a full spectrum of functioning. PROMIS investigators utilized standardized procedures to create item banks in many domains including emotional distress, fatigue, pain, physical functioning, and peer relationships [12]. The item banks have undergone extensive psychometric evaluation including assessment of validity and reliability [13–16]. For example, the pediatric item banks have been validated in general populations and in children with sickle cell disease, obesity, cancer, rheumatic diseases, chronic kidney disease, and rehabilitative needs. Item banks can be administered as computer adaptive tests (CATs) or fixed length short forms with high relative validity across a broad range of functioning. Additionally, item banks can be used to construct custom short forms. Because PROMIS item banks were evaluated using item response theory (IRT) [17], scores from any subset of bank items (e.g., custom short form, CAT) produce standardized scores on the same scale, regardless of which items from the bank are included in the measure. This enables construction of a custom short form that specifically targets lower levels of physical functioning that would be hypothesized to better distinguish patients in this range of functioning.

The purpose of this study was to create two custom parent-proxy physical functioning short forms using the PROMIS item banks in Upper Extremity function and Mobility and explore scores and preliminary psychometric properties among children with chronic respiratory insufficiency due to diverse underlying neuromuscular illnesses that put them at risk for low levels of physical functioning. We assessed validity by comparing to scores on physical and mental health items from the Child Health Rating Inventories (CHRIs) global health scale. Given the cognitive, communication, and functional limitations in this population of children, we relied on parent-proxy report. Although agreement between child self-report and parent-proxy report is not always high for domains that are “beneath the skin,” such as emotional or social functioning, there is generally better agreement on more objective domains, such as physical functioning [18, 19].

## Methods

### Sample selection

The Critical Care, Anesthesia, Perioperative Extension (CAPE) and Home Ventilation Program at Boston Children’s Hospital provides care coordination to pediatric patients with risk for chronic respiratory insufficiency from a variety of underlying chronic illnesses, including muscular and neuromuscular disorders. As part of a larger effort to understand the impact of these conditions on children’s functioning and well-being, parents were invited to participate in an HRQL study, described elsewhere [20]. Briefly, patients, ages 30 days to 22 years, who were receiving ongoing care from the CAPE Program and not living in a residential facility were eligible. Parent caregivers had to be at least 18 years old and actively participate in their child’s care. Of the 197 parents eligible for screening as of March 31, 2013, 140 parents were enrolled in the HRQL cohort. This study was approved by the Institutional Review Board at Boston Children’s Hospital.

We evaluated a subset of parents in the HRQL cohort who had children ≥5 years old (*n* = 70) because PROMIS parent-proxy reported items were developed and validated for this age range. A subset of children who were chronologically ≥5 years old, but had intellectual disabilities based on prior clinical evaluation and whose parents were completing the measures online were excluded. Although outside the validation age range, we also included parent-proxy report of patients up to 22 years old because their underlying diseases often cause cognitive, communication, and functional limitations that differentiate young adults with chronic respiratory insufficiency from their typically developing peers who are transitioning to adulthood between ages 18 and 22. Of note, the CHRIs Global measure has been validated in parent proxies of patients 5–21 years. This resulted in 57 participants in the present analysis.

### Measures

Parent participants completed measures about both their own HRQL and their child’s HRQL every 6 months for up to four time periods. However, only the first completed assessment was used in this analysis. Participants could complete measures either on paper or online via StudyTRAX (Macon, GA), a web-based data collection platform (http://www.studytrax.com/).

#### Custom PROMIS parent-proxy Upper Extremity and Mobility short forms

Two new custom parent-proxy short forms were created for this study using two PROMIS physical functioning item banks (v1.0): (1) Upper Extremity (13 items selected from 29) and (2) Mobility (13 items selected from 24) [15, 16, 21]. All items use a 7-day recall period. A multi-disciplinary team, including two pediatric subspecialty physicians with more than 15 years’ experience with similar patient populations (SKP and RJG), and two PROMIS investigators (NER and DAD), provided their expertise when selecting items for the custom short forms. The following criteria were used: ability to capture a range of physical functioning activities, reflecting the full spectrum of neuromuscular activities (i.e., distal-proximal); relevance to activities of daily activity (e.g., eating, bathing, dressing); elimination of potentially insensitive items (e.g., items asking parents to compare their child’s physical functioning to other children their age); and avoidance of redundant items. Item difficulties (theta), a metric used to determine the place on the latent trait that the item provides the most information about individual differences [22], were provided by PROMIS and were used to identify items that capture scores at the lower end of the scale. In addition, given the wide variation in these children’s ability to ambulate due to their underlying conditions, questions about the use of assistive devices were included. Item responses had five levels that ranged from 0 = “not able to do” to 4 = “with no trouble,” with the exception of the two assistive technology questions which ranged from 4 = “almost always” to 0 = “never.” Some response options were collapsed during scoring based on instructions from measure developers.

PROMIS investigators produced raw score to T-score look-up tables specifically for the two custom short forms based on item parameters. The T-score look-up tables were computed using the well-validated summed score Lord-Wingersky recursive algorithm [23], extended to polytomous items [24]. For each summed score, the algorithm computes the most likely T-score estimate and standard error (SE). Although it is possible that the look-up table approach would create larger SEs than direct IRT-based scoring, these differences are cancelled out in group-level analysis. Further, the larger number of items in each custom short form compared to the standard PROMIS short forms (8 items) uniformly reduces SE across the score continuum, resulting in a high level of reliability. The T-scores for both custom short forms were centered on the PROMIS pediatric calibration population with a mean of 50 and a standard deviation (SD) of 10, where higher scores represent better functioning [15, 16]. IRT-based scoring, including the use of T-score look-up tables, allows comparisons to scores from other samples, even when they have answered different questions from the item banks. The Upper Extremity short form T-scores had a possible range of 11.5 to 52.5, while the Mobility short form T-scores had a possible range of 12.8 to 54.7.

#### Child Health Rating Inventories (CHRIs) Global Health scale

The Global Health scale from the CHRIs-General, [5, 25, 26] a validated and reliable generic HRQL tool with parent and child versions, was used to assess HRQL. As part of the CHRIs Global Heal﻿th scale, parents completed seven individual items on the child’s global HRQL, including single, summary items on overall mental health and on overall physical health. Like the PROMIS measures, these items also use a 7-day recall period. Each item has a 5-level response set that ranges from “poor” to “excellent,” but was collapsed to three levels (poor/fair, good, very good/excellent) because of the limited sample size and the need to create known groups of sufficient size for comparison.

#### Demographic and clinical variables

At baseline, parents provided data about the following child and family demographic characteristics: child gender, child age, parent gender, parent age, and parent education. The child’s race/ethnicity and insurance was supplied by clinical staff along with the following baseline clinical information: diagnosis, respiratory support status, and physician-rated clinical severity (1 = least severe, 10 = most severe), the latter adapted from a validated single item severity measure from the National Survey of Children with Special Health Care Needs that reflects judgment about likelihood of clinical complications [27].

### Statistical analysis

Demographic and clinical variables were summarized using means (SDs) or medians (25th–75th percentiles) for continuous variables or using frequencies and percentages for categorical variables.

#### Psychometric analysis

The frequency and percentages of participants in each response category of the Upper Extremity and Mobility items were reported to demonstrate the distribution. The percentage responding at the lowest and highest response category can be considered the floor and ceiling percentage, respectively. Means, SDs, and missingness were calculated for each raw item score and for the Upper Extremity and Mobility summary T-scores. For the Upper Extremity and Mobility summary T-scores, we reported the percentage at the measurement floor and ceiling (i.e., the highest and lowest scores based on the possible range). Ceiling and floor effects are considered moderate when >15% [28]. Known groups comparisons were made for the physician-rated clinical severity item (split into two groups at the median) and the CHRIs physical health and mental health items (collapsed to three levels). The PROMIS physical function T-scores were compared using the Wilcoxon rank sum test for the clinical severity item and Spearman correlation for the CHRIs physical and mental health items. Although there are no established criteria for the interpretation of correlations to measure concurrent validity, correlations of <0.29 are generally considered low, 0.30–0.60 are considered moderate, and >0.60 are considered high [29]. All analyses were conducted in SAS version 9.2 (SAS Institute, Inc., Cary, NC); the alpha level was set at 0.05.

## Results

The custom Upper Extremity and Mobility short forms were completed by 57 parent proxies. The mean child age was 12 years (SD = 6) and nearly half were female (Table 1). The majority of patients (58%) had both private and public insurance to cover their health care needs. SMA was the most common diagnosis (35%) and the median clinical severity was 6 (possible range 1 to 10). Most patients had some degree of respiratory support; 40% had artificial respiratory and ventilator support, while 32% had non-invasive respiratory support. The mean parent age was 44 (SD = 8), most were female (83%), and most were college graduates (63%).

**Table 1:** Baseline Demographic and Clinical Characteristics, *n* = 57

Child Characteristics	
Child age, mean (SD)	12.4 (6.2)
Child female, n (%)	28 (49.1%)
Race/ethnicity, n (%)	
White, non-Hispanic	42 (73.7%)
Asian	6 (10.5%)
Black, non-Hispanic	3 (5.3%)
Hispanic/Latino	3 (5.3%)
Unknown	3 (5.3%)
Insurance, n (%)	
Private only	11 (19.3%)
Public only	13 (22.8%)
Private and public	33 (57.9%)
Disease Characteristics	
Diagnosis, n (%)	
Acquired injury	6 (10.5%)
Anomalies (All)	4 (7.0%)
Chronic lung disease	4 (7.0%)
Dystrophies	12 (21.1%)
SMA (Types I, II, III)	20 (35.1%)
Other	11 (19.3%)
Respiratory support, n (%)	
Artificial	3 (5.3%)
Artificial + ventilator	23 (40.4%)
Non-invasive	18 (31.6%)
None	13 (22.8%)
Physician-rated clinical severity, median (25th–75th percentile)	6.0 (4.0–7.0)
Family Characteristics	
Parent female, n (%)	47 (82.5%)
Parent age, mean (SD)	43.7 (7.8)
Parent education, n (%)^a^	
< High school	2 (3.7%)
High school graduate	6 (11.1%)
Some college	12 (22.2%)
≥ College graduate	34 (63.0%)

^a^Four parents did not complete the education question

*SMA* spinal muscular atrophy

The percentage of parents endorsing the most severe response option (i.e., “not able to do”) varied by item (Table 2). For example, among the Upper Extremity items, 30% answered that the child could not move their hands or fingers, while 67% reported that the child could not pull a shirt over his/her head. Among the Mobility items, 27% of parents reported that the child could not turn his/her head all the way to the side, while 68% could not get up from a regular toilet. There were <2% missing data for any item. The mean Upper Extremity T-score was 21.4 (SD = 12.6) and the mean Mobility T-score was 22.0 (SD = 11.1; Table 3). For the Upper Extremity scale, 26.3% scored at the measurement floor and 10% scored at the measurement ceiling, while 15.8% scored at the measurement floor and 5.3% at the measurement ceiling for the Mobility scale.

**Table 2:** Frequencies and percentages of responses to Upper Extremity and Mobility items

	N	Not able to do	With a lot of trouble	With some trouble	With a little trouble	With no trouble
Upper Extremity Items						
Pull shirt over head	57	38 (66.7%)	3 (5.3%)	2 (3.5%)	5 (8.8%)	9 (15.8%)
Put on shoes	57	43 (75.4%)	0 (0%)	3 (5.3%)	3 (5.3%)	8 (14.0%)
Use key to unlock door	57	42 (73.7%)	3 (5.3%)	1 (1.8%)	1 (1.8%)	10 (17.5%)
Zip up clothes	57	40 (70.2%)	3 (5.3%)	1 (1.8%)	3 (5.3%)	10 (17.5%)
Put toothpaste on toothbrush	57	37 (64.9%)	5 (8.8%)	2 (3.5%)	2 (3.5%)	11 (19.3%)
Put on clothes without help	57	41 (71.9%)	2 (3.5%)	4 (7.0%)	1 (1.8%)	9 (15.8%)
Put on socks without help	57	41 (71.9%)	1 (1.8%)	4 (7.0 %)	5 (8.8%)	6 (10.5%)
Open clothing drawers	57	35 (61.4%)	5 (8.8%)	1 (1.8%)	4 (7.0%)	12 (21.1%)
Hold a full cup	57	31 (54.4%)	2 (3.5%)	4 (7.0%)	4 (7.0%)	16 (28.1%)
Use a mouse or touch pad ^a^	56	18 (32.1%)		4 (7.1%)	5 (8.9%)	29 (51.8%)
Wash face with cloth ^b^	57	38 (66.7%)			6 (10.5%)	13 (22.8%)
Move hands or fingers ^a^	57	17 (29.8%)		3 (5.3%)	9 (15.8%)	28 (49.1%)
Write with pen or pencil ^a^	57	22 (38.6%)		8 (14.0%)	5 (8.8%)	22 (38.6%)
Mobility Items						
Get up from the floor	57	39 (68.4%)	2 (3.5%)	6 (10.5%)	4 (7.0%)	6 (10.5%)
Move legs	57	15 (26.3%)	11 (19.3%)	8 (14.0%)	8 (14.0%)	15 (26.3%)
Stand up without help	57	38 (66.7%)	1 (1.8%)	1 (1.8%)	5 (8.8%)	12 (21.1%)
Walk up stairs without holding on	57	45 (79.0%)	5 (8.8%)	2 (3.5%)	2 (3.5%)	3 (5.3%)
Get into bed	56	40 (71.4%)	0 (0%)	2 (3.6%)	3 (5.4%)	11 (19.6%)
Walk across room	57	38 (66.7%)	0 (0%)	1 (1.8%)	3 (5.3%)	15 (26.3%)
Bend over to pick something up	57	41 (71.9%)	0 (0%)	2 (3.5%)	7 (12.3%)	7 (12.3%)
Walk more than one block	57	43 (75.4%)	5 (8.8%)	0 (0%)	4 (7.0%)	5 (8.8%)
Get up from regular toilet	57	39 (68.4%)	1 (1.8%)	2 (3.5%)	3 (5.3%)	12 (21.1%)
Get down on knees without holding on	57	46 (80.7%)	0 (0%)	2 (3.5%)	5 (8.8%)	4 (7.0%)
Turn head all the way to the side	56	3 (5.3%)	0 (0%)	1 (1.8%)	2 (3.5%)	51 (89.5%)
	N	Almost always	Often	Sometimes	Almost never	Never
Used a wheelchair ^c^	56	15 (26.8%)	8 (14.3%)	4 (7.1%)	11 (19.6%)	18 (32.1%)
Used a walker, cane, or crutches ^c^	57	32 (57.1%)	3 (5.4%)	7 (12.5%)	4 (7.1%)	10 (17.9%)

^a^Response levels “with a lot of trouble” and “not able to do” collapsed per PROMIS scoring algorithm

^b^Response levels “with some trouble,” “with a lot of trouble,” and “not able to do” collapsed per PROMIS scoring algorithm

^c^Not used in Mobility T-score because item did not perform well

**Table 3:** Raw item scores and Upper Extremity and Mobility T-scores

	N	Mean (SD)	Median (25th–75th percentile)	Min, Max
Upper Extremity				
T-score	57	21.4 (12.6)	16.9 (11.5, 22.5)	11.5, 52.5
Pull shirt over head	57	1.0 (1.6)	0 (0, 2)	0, 4
Put on shoes	57	0.8 (1.5)	0 (0, 0)	0, 4
Use key to unlock door	57	0.8 (1.6)	0 (0, 1)	0, 4
Zip up clothes	57	0.9 (1.6)	0 (0, 1)	0, 4
Put toothpaste on toothbrush	57	1.0 (1.6)	0 (0, 2)	0, 4
Put on clothes without help	57	0.9 (1.5)	0 (0, 1)	0, 4
Put on socks without help	57	0.8 (1.4)	0 (0, 2)	0, 4
Open clothing drawers	57	1.2 (1.7)	0 (0, 3)	0, 4
Hold a full cup	57	1.5 (1.8)	0 (0, 4)	0, 4
Use a mouse or touch pad ^a^	56	1.8 (1.4)	3 (0, 3)	0, 3
Wash face with cloth ^b^	57	0.6 (0.8)	0 (0, 1)	0, 2
Move hands or fingers ^a^	57	1.8 (1.3)	2 (0, 3)	0, 3
Write with pen or pencil ^a^	57	1.5 (1.4)	1 (0, 3)	0, 3
Mobility				
T-score	57	22.0 (11.1)	16.7 (14.5, 29.7)	12.8, 54.7
Get up from the floor	57	0.9 (1.4)	0 (0, 2)	0, 4
Move legs	57	1.9 (1.6)	2 (0, 4)	0, 4
Stand up without help	57	1.2 (1.7)	0 (0, 3)	0, 4
Walk up stairs without holding on	57	0.5 (1.1)	0 (0, 0)	0, 4
Get into bed	56	1.0 (1.7)	0 (0, 2.5)	0, 4
Walk across room	57	1.2 (1.8)	0 (0, 4)	0, 4
Bend over to pick something up	57	0.9 (1.5)	0 (0, 2)	0, 4
Walk more than one block	57	0.6 (1.3)	0 (0, 0)	0, 4
Get up from regular toilet	57	1.1 (1.7)	0 (0, 3)	0, 4
Get down on knees without holding on	57	0.6 (1.3)	0 (0, 0)	0, 4
Turn head all the way to the side	56	2.2 (1.6)	3 (0, 4)	0, 4
Used a wheelchair ^c^	56	1.2 (1.6)	0 (0, 2.5)	0, 4
Used a walker, cane, or crutches ^c^	57	3.7 (0.9)	4 (4, 4)	0, 4

^a^Response levels “with a lot of trouble” and “not able to do” collapsed per PROMIS scoring algorithm

^b^Response levels “with some trouble,” “with a lot of trouble,” and “not able to do” collapsed per PROMIS scoring algorithm

^c^Not used in Mobility T-score because item did not perform well

With regards to assistive devices, more than half reported that their child always used a wheelchair to get around. Nearly 90% reported that their child never used a walker, cane, or crutches to get around, but this likely reflects their inability to use these devices rather than their ability to walk. As such, these two items on assistive devices were excluded from the Mobility scoring algorithm (resulting in an 11-item scale).

**Fig. 1:**
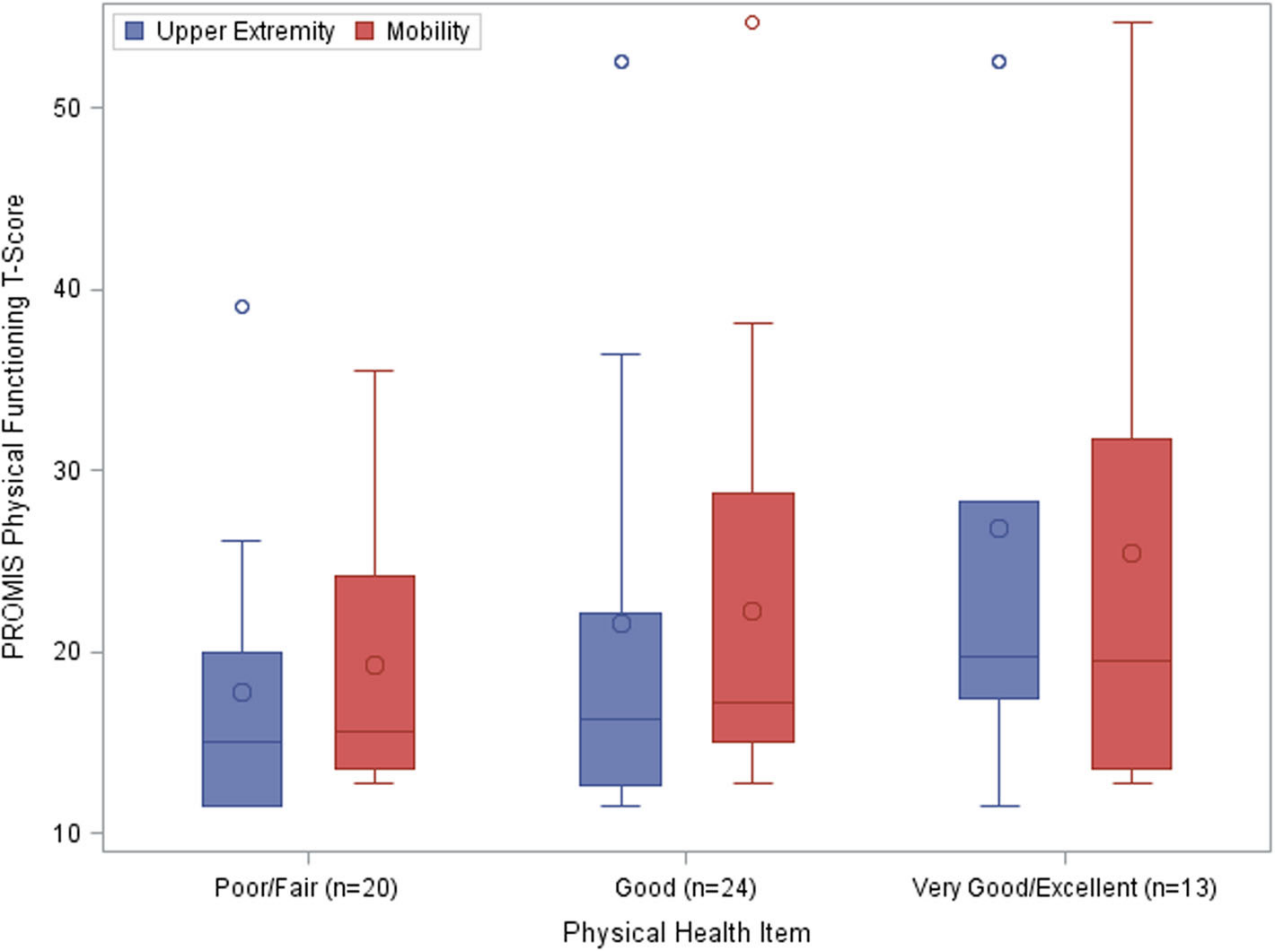
Boxplots of PROMIS physical function T-scores by physical health item score. Spearman correlation for Upper Extremity: *r* = 0.28 (*p* = 0.04); Spearman correlation for Mobility: *r* = 0.15 (*p* = 0.26). Note: the line within the box represents the median; the circle within the box represents the mean; the length of the box represents the interquartile range; the length of the whiskers represents the distance between the box and the observation that is less than 1.5 times the interquartile range; the points outside the whiskers represent outliers

**Fig. 2:**
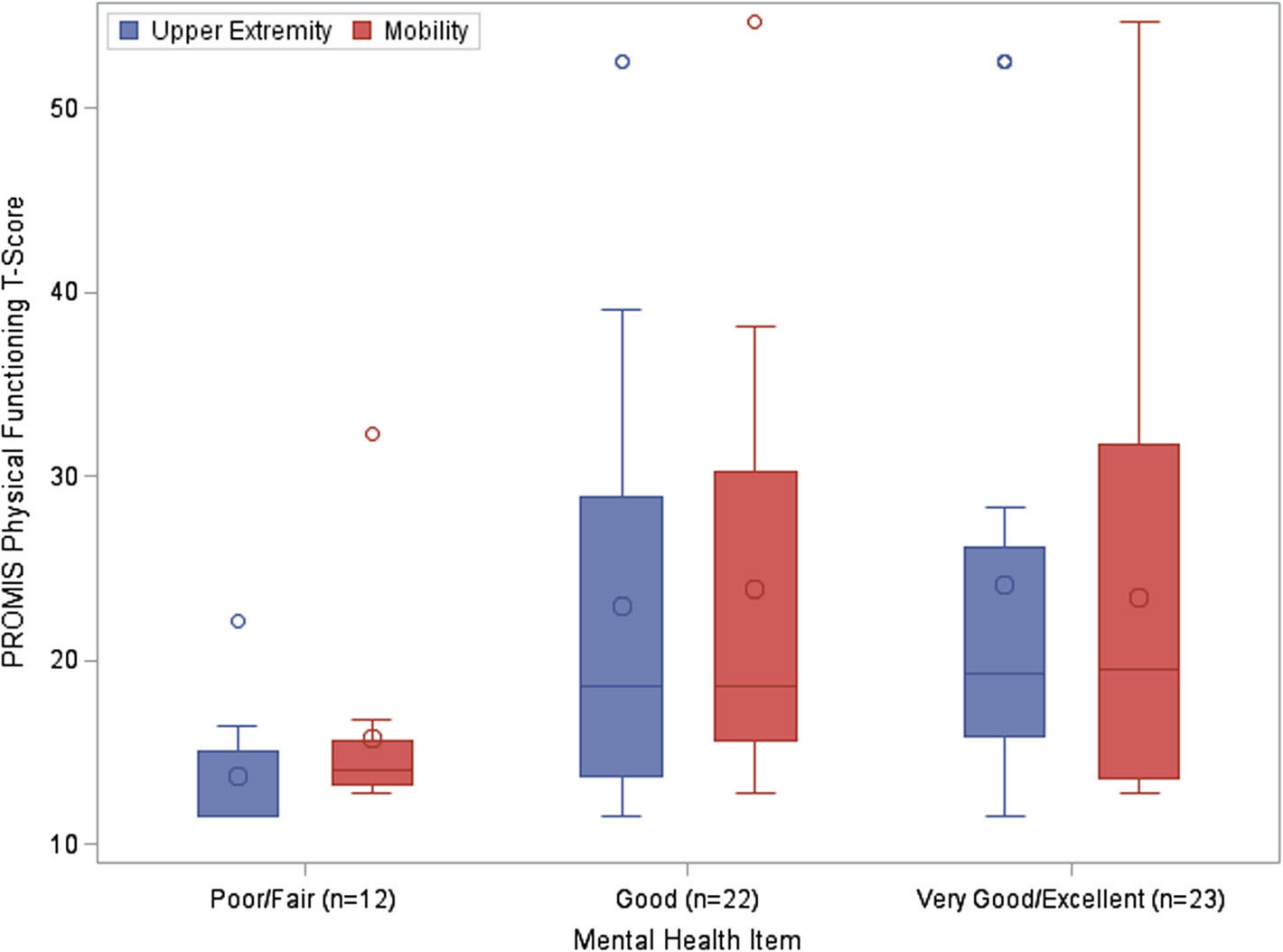
Boxplots of PROMIS physical function T-scores by mental health item score. Spearman correlation for Upper Extremity: *r* = 0.35 (*p* = 0.008); Spearman correlation for Mobility: *r* = 0.21 (*p* = 0.12). Note: the line within the box represents the median; the circle within the box represents the mean; the length of the box represents the interquartile range; the length of the whiskers represents the distance between the box and the observation that is less than 1.5 times the interquartile range; the points outside the whiskers represent outliers

When physician-rated clinical severity was split at its median (6), the median Upper Extremity T-score for the less severe group was significantly higher (21.8; 25th–75th percentile: 17.9, 28.9) than the T-score in the more severe (14.1; 25th–75th percentile: 11.5, 18.6; *p* < 0.001). Similarly, the median Mobility T-score for the less severe group was significantly higher (27.8; 25th–75th percentile: 16.7, 35.5) than the T-score in the more severe group (15.6; 25th–75th percentile: 13.6, 19.5; *p* = 0.004). Both Upper Extremity and Mobility T-scores were slightly higher in the subgroups defined by better CHRIs physical health item scores (Fig. 1; *r* = 0.28 (*p* = 0.04), *r* = 0.15 (*p* = 0.26), respectively). Upper Extremity and Mobility T-scores also were slightly higher among those with better CHRIs mental health item scores (Fig. 2; *r* = 0.35 (*p* = 0.008), *r* = 0.21 (*p* = 0.12), respectively). Figure 1 shows that the relationship between Upper Extremity and Mobility T-scores appear more linear for the physical health item (T-scores are progressively higher for those scoring good and very good/excellent, compared to poor/fair). In contrast, Fig. 2 shows that the Upper Extremity and Mobility T-scores are higher for those scoring good on the mental health item compared with poor/fair, but there is little difference between the good and very good/excellent categories.

## Discussion

Custom parent-proxy PROMIS short forms in Upper Extremity and Mobility were created and administered to 57 parents of children with chronic respiratory insufficiency, secondary to a variety of disorders, including neuromuscular illnesses. The creation of these custom short forms was made possible by the previously validated parent-proxy PROMIS item banks and scoring based on IRT models. Mean T-scores confirmed that physical functioning in this sample was severely affected with scores nearly three standard deviations below the PROMIS pediatric calibration population mean. Even among children in the less severe group, mean T-scores were at least two standard deviations below the calibration population. Preliminary psychometric properties demonstrated that there were multiple items in the PROMIS banks that targeted lower levels of functioning and the potential of the two custom short forms to more accurately measure physical functioning in those at risk for lower levels of functioning. However, there were some participants still scoring at the floor of the scale despite our targeting items at the lower end of functioning.

The custom short forms demonstrated known groups validity with patients with worse clinical severity having lower Upper Extremity and Mobility T-scores, as expected. There were low to moderate correlations between the Upper Extremity and Mobility T-scores and the CHRIS-General items for physical and mental health. Given our hypothesis that the physical functioning scores would be more highly correlated for the physical health item than the mental health item, we were surprised to find only low to moderate correlations and that the strength of the correlation was slightly higher for the mental health item. However, the data demonstrated a weak linear relationship in the Upper Extremity and Mobility T-scores for better physical health item scores, while the Upper Extremity and Mobility T-scores showed a threshold effect for mental health item scores of good or very good/excellent compared to poor/fair. This may imply that HRQL within the domain of mental health is most severely impacted for those with the worst physical functioning. Additionally, the use of the parent-proxy rater may also explain the stronger relationship with mental health. As an example, the parent may observe that the child is unable to do many of the physical functioning items and assume these physical limitations would adversely affect the child’s mental health and well-being. Future research could assess item ordering and whether including physical functioning items before the mental health item results in lower mental health scores. Given the parents’ report of association between children’s physical functioning and their mental health, it would be useful in future studies to collect information from the child participants about their own mental health. In parallel, as has been reported in other serious illness, it would be important to assess the impact of the child’s condition on the parents’ mental health—either by incorporating questions from PROMIS Mental Health item banks or established short forms or qualitative work with parents to help us better understand this relationship.

With a mean of 50 in the PROMIS pediatric calibration population, the range of possible T-scores for each custom short form (11.5 – 52.5 for Upper Extremity; 12.8 – 54.7 for Mobility) demonstrates that the items targeted the lower range of physical function. Additionally, the custom PROMIS short forms were able to capture differences at the lower end of physical functioning. However, there is evidence that some participants still scored at the floor of the scales. For the Upper Extremity scale, 26% scored at the measurement floor and for five out of 13 items more than 70% of parent-proxy raters scored their child at the lowest response category. Similarly, 16% scored at the floor for the Mobility scale and for five of the 11 items more than 70% of parent-proxy raters scored their child at the lowest response category. The use of IRT when scoring PROMIS measures should help to overcome floor/ceiling effects, but the persistence of children at the measurement floor indicates that for those at the lowest levels of physical functioning, these specific items are not sufficient for distinguishing their level of functioning. To address this, additional items at the lowest end of the scale may need to be developed [30] or “borrowed” from other scales, such as the Pediatric Neuro-QOL [31], and calibrated to this population to supplement the existing item bank. The Pediatric Neuro-QOL was developed for use in children with neurological conditions who experience lifelong functional limitations, including those with muscular dystrophy, demonstrating the relevance of their target population to the current study population. Of interest, responses in our study were primarily at the floor or ceiling of items, rather than in the middle categories. The number at the ceiling may reflect our targeting of the items at the lower end of the scale. However, it may also indicated there are two heterogeneous groups for which only some need to receive the custom parent-proxy short forms; perhaps a screener item could help differentiate the groups. Additionally, further analysis with larger sample sizes using IRT is needed to address the potential local dependence among some of the items near the lower end of the scale. Local dependence occurs when items in a scale are related to each other. Following testing in a larger group of children, we may also remove some of the items with large floor effects to improve the measurement properties.

The current study describes the development of custom PROMIS short forms targeted to a population of children with respiratory insufficiency and resulting decreased physical functioning. CATs, which tailor items to administer based on one’s responses to previous items, are another powerful tool that could be used to measure physical functioning in this population. However, the goal of existing CATs, based on the PROMIS item banks, is to arrive at a T-score with a certain level of precision for the majority of respondents [32]. To be useful in patients with severe functional loss, the existing item bank may need to be enriched with newly calibrated items at the lower end of the scale, as indicated by the percentage of children scoring at the measurement floor on the custom short forms.

The two questions about assistive devices included as part of the Mobility short form proved to be inadequate in this population of children who are heavily reliant on these devices. For some children, self-propelled or electric wheelchairs are introduced to help overcome weakness, prevent fatigue, and preserve participation in role activities (e.g., school). In contrast, for children with severe perinatal injury or abrupt spinal injury, “wheelchairs” often resemble more of a stroller or stretcher for transport and still require assistance from a caregiver. Within our sample, more than half of children used a wheelchair almost always while less than 20% never required a wheelchair. In contrast, 90% of children never used a walker, cane, or crutches. In a general population, never using these devices indicates good physical functioning. However, in this population, never using these devices more likely represents an inability to use them. Further modification of assistive devices questions are needed for use in this population with attention paid to types of devices needed, similar to the work that been done with adults requiring assistive devices [33, 34]. In addition, the evolution of new adaptive equipment, including eye gaze technology, exoskeletons and bionics, will require further considerations for questions with and without devices or supports. Eye gaze technology, for example, takes advantage of motion sensors calibrated to eye movements that permit a person to interface with communication devices or electronic platforms that greatly enhances a person’s ability to manipulate the environment, but may not alter their physical functioning.

Within clinical practice, the physical functioning short forms can provide the medical team with additional information about how the child’s condition is affecting their functioning. Parents or children could also complete the forms between clinic visits to allow for remote monitoring of the child’s condition that could signal deterioration requiring clinical intervention. This remote data collection would not be possible for the clinical measures that require clinician involvement [7–11]. Given the different expected rates of disease progression based on the underlying diagnosis, [35, 36] collecting physical functioning scores over time could provide useful information. Deteriorating scores could indicate the need for medical or surgical intervention. Scores could also be used to assess the effectiveness of new treatments, such as gene-targeted therapies in SMA, or assess functional outcomes that may be more meaningful to the child and family than results of clinical tests, such as nerve conduction, electromyographs, and serum protein analyses.

The items within each of the custom short forms also provide information that can help to allocate needed services. For example, children requiring help getting dressed, getting into bed, and using the toilet typically require around-the-clock care. Adding a home health aide or extending home nursing hours may help address some of these needs, and remove some of the burden on the parent caregivers. In addition, parent caregivers of children requiring near constant care may require additional emotional support [37–39]. Further, questions about using a mouse or touch pad recognize the role technology can play in improving communication and independence within this population. If children indicate that they are capable of using a mouse or touchpad, self-report of these short forms and other HRQL measures may be possible. Similarly, additional items about eye gaze technology could also help us to better understand communication abilities and those children capable of providing self-report with assistance.

Future studies that include both clinical assessment and self- (or parent-proxy) reported HRQL are necessary to further understand the relationship between these measures and to further establish validity of the custom PROMIS short forms. For example, the Pediatric Evaluation of Disability Inventory-Computer Adaptive Test (PEDI-CAT) is a parent-completed measure that includes domains on daily activities, mobility, social/cognitive functioning, and responsibility [40]. Unlike some other generic HRQL tools, the PEDI-CAT contains items that capture a range of functional levels, including the lower end of mobility [41]. However, the PEDI-CAT cannot be completed remotely between clinic visits because it is not web-based. Future studies are planned to compare mobility scores from the PEDI-CAT and the custom PROMIS short forms to better understand the validity.

We acknowledge the study’s limitations. First, this is a relatively small sample of parent-proxy respondents. However, the goal was to explore scores and preliminary psychometrics of these two custom short forms rather than to generalize findings to a larger population. Second, this is a heterogeneous sample with various underlying diagnoses with different effects on physical functioning and the physical functioning trajectory. Given that these diagnoses are relatively rare within an institution, and are often cared for together in multi-disciplinary clinics, the heterogeneous sample reflects the clinical reality. However, a larger sample is needed to better explore differences within and across diagnoses. Third, due to the cognitive, communication, and functional limitations in this sample, we relied on parent-proxy report rather than child self-report and did not develop custom physical functioning self-report forms in parallel. Related to this, we included parent-proxy report of patients up to 22 years old, which is outside the measure validation age range, because their underlying disease causes limitations that differentiate young adults with chronic respiratory insufficiency from their typically developing peers. Future research is needed to understand differences in parent-proxy report and child self-report, where possible, particularly among those children who could complete self-report measures with communication aids. Rater differences may help explain the stronger observed relationship between physical functioning and mental health than physical health based on parent-proxy report as discussed above. Fourth, we did not have any objective measures of physical functioning in this population, such as specific clinical assessments, with which to validate the parent-proxy ratings of HRQL.

## Conclusion

In conclusion, we found that children with chronic respiratory insufficiency due to neuromuscular illnesses had low mean Upper Extremity and Mobility T-scores, compared with the PROMIS calibration norms. There was evidence of known groups validity, especially compared with physician-rated clinical severity. However, floor effects still existed for some participants and items about assistive devices did not perform well in this group. Additional data collection is planned to further test the performances of the custom PROMIS short forms in larger patient cohorts using IRT and adding newly calibrated items that may help discriminate at the very low end of physical functioning.

## Abbreviations

CAPE: Critical Care, Anesthesia, Perioperative Extension
CAT: Computer adaptive tests
CHRIs: Child Health Rating Inventories
HRQL: Health-related quality of life
MD: Muscular Dystrophy
PedsQL™: Pediatric Quality of Life Inventory
PROMIS™: Patient Reported Outcomes Measurement Information System
SMA: Spinal muscular atrophy
